# Psychometric Evaluation of a Patient-Reported Symptom Index for Nonmuscle Invasive Bladder Cancer: Field Testing Protocol

**DOI:** 10.2196/resprot.8761

**Published:** 2017-11-08

**Authors:** Claudia Rutherford, Madeleine T King, David P Smith, Daniel SJ Costa, Margaret-Ann Tait, Manish I Patel, NMIBC-SI Working Group

**Affiliations:** ^1^ School of Psychology The University of Sydney Sydney Australia; ^2^ Sydney Medical School The University of Sydney Sydney Australia; ^3^ Cancer Research Division Cancer Council NSW Kings Cross Australia; ^4^ Pain Management Research Institute Royal North Shore Hospital St Leonards Australia; ^5^ Nonmuscle Invasive Bladder Cancer Symptom Index Working Group Sydney Australia

**Keywords:** quality of life, patient reported outcome measures, cancer, bladder cancer, surveys and questionnaires

## Abstract

**Background:**

Nonmuscle invasive bladder cancer (NMIBC) is a chronic condition requiring intensive follow-up, repeated endoscopic examinations, tumor resections, and intravesical treatments that can occur every 3 months for life. In this clinical context, patient-reported outcomes (PROs) are a critical concern for patients and their managing clinicians. PROs have enormous potential to be integral to treatment assessment and recommendations for NMIBC; however, current PRO measures are inadequate for NMIBC because they lack key NMIBC-specific symptoms and side effects associated with contemporary treatments.

**Objective:**

The overarching aim of this study was to develop and evaluate a patient-reported symptom index (SI) for individuals with NMIBC (the NMIBC-SI) that is acceptable to patients; reliable, valid, and responsive to differences between contemporary treatments for NMIBC; and fit for purpose as an endpoint in clinical trials.

**Methods:**

The NMIBC-SI will be evaluated in 2 field tests across a total of 3 years. Field test 1 is a cross-sectional study design involving 225 adult NMIBC patients recruited while undergoing active treatment or those who completed final treatment within the past week. Data collected include patient demographics, clinical features of the tumor, risk category, treatment type, comorbidity, and PROs. Field test 2 is a prospective longitudinal study involving 225 newly diagnosed NMIBC-SI patients. Clinical data and patient-completed questionnaires will be collected at 4 time points during treatment: before tumor resection, 1 week after resection, end-of-induction intravesical therapy, and 1-year follow-up. Standard psychometric tests will be performed to assess the reliability, validity, responsiveness, and clinical utility of the NMIBC-SI.

**Results:**

Participant recruitment to field test 1 commenced in February 2017. Recruitment for field test 2 is planned to commence in January 2018. Final results are expected to be published in 2019. The NMIBC-SI will be freely available for use via registration.

**Conclusions:**

This study protocol contains detailed methods that will be used across multiple international sites. Phase 2 in the development of the NMIBC-SI will enable a comprehensive evaluation of its reliability, validity, and responsiveness to ensure that the NMIBC-SI is fit for purpose in clinical research and provides an evidence base for the ongoing improvement of future therapies for NMIBC.

**Trial Registration:**

ClinicalTrials.gov NCT03091764; http://clinicaltrials.gov/ct2/showNCT03091764 (Archived by WebCite at http://www.webcitation.org/6umBhQeNX)

## Introduction

### Nonmuscle Invasive Bladder Cancer Is a Chronic Health Problem

Bladder cancer is the 9th most common cancer diagnosed worldwide and the 13th most common cause of cancer death [1]. Internationally, 430,000 new cases of bladder cancer were diagnosed in 2012, with 165,000 recorded deaths [2]. In Australia alone, in 2014, an estimated 2730 people were diagnosed with bladder cancer [3], and there were 1040 recorded deaths from it [1,4]. Bladder cancer is 3 to 4 times more prevalent in males than in females, and incidence increases with age, with people older than 55 years accounting for 90% of new diagnoses [5]. Around 75% of bladder cancer diagnoses are not muscle invasive at first diagnosis [6]. Because 5-year survival in this group exceeds 80% [7], the prevalence of nonmuscle invasive bladder cancer (NMIBC) is 10 times its incidence, even though the overall rate of recurrence is approximately 60% to 70% and progression is 20% to 30% [8].

Nonmuscle invasive bladder cancer is a chronic disease. Its management depends on the risk of the bladder cancer recurring and progressing [6]: low-risk patients receive frequent cystoscopies, possible tumor resections, and single instillations of postoperative chemotherapy; intermediate-risk patients usually require intravesical therapy, which lasts between 6 weeks and 3 years; and high-risk patients require intravesical Bacillus Calmette–Guérin (BCG; immunotherapy), which starts with 6-week induction treatment and continues with maintenance for 1 to 3 years. The higher the risk, the more the requirement for intravesical therapy. Follow-up is mandatory, with repeated endoscopic examinations, radiological imaging, biopsies, and tumor resections as frequent as 3-monthly and life-long. This makes the cost per patient from diagnosis to death the highest of all cancers [9], contributing a major economic and resource burden on health care systems [9].

Guidelines for the management of NMIBC are based on evidence for the effectiveness of treatments in reducing risk of bladder cancer progression and recurrence. Evidence for reductions in health-related quality of life (HRQoL) and associated side effects with each treatment are not incorporated into the decision-making process because they have been poorly documented (see section below on Critical Gaps in Knowledge About HRQoL and Patient-Reported Outcomes [PROs] in NMIBC). This is despite the fact that these treatments can cause substantial side effects and local and systemic toxicity. For example, the commonly used BCG has proved effective in reducing recurrences in patients with high-grade tumors and carcinoma in situ, but it can cause moderate to severe local and systemic side effects, and only 16% are able to complete their full treatment schedule [10].

### Why Patient-Reported Outcomes Are Important

A PRO is any report that comes directly from patients about how they feel in relation to their health condition and its therapy, without interpretation by others [11]. PROs can include symptoms, function, HRQoL, and perceptions of treatment. These patient reports are captured and quantified by patient-reported outcome measures (PROMs) in the form of questionnaires.

PROs are beneficial for improving patient-clinician communication, prioritizing patient-centered care, and improving service provision [12,13]. In contrast to life-threatening conditions where survival endpoints may dominate HRQoL considerations, PROs should be key considerations in chronic conditions [14]. For example, a treatment found to be effective in improving survival in a clinical trial may fail in the real world because of toxicity and reduced HRQoL, which could compromise compliance and subsequently its effectiveness [15]. Similarly, the inconvenience of repeated clinic visits for monitoring or treatment can compromise compliance [16]. The acceptance of PROM-based evidence by regulatory bodies is reflected in the US Food and Drug Administration’s (FDA) approval of PROs to support product labeling claims [17].

### Critical Gaps in Knowledge About HRQoL and PROs in NMIBC

The primary goal in managing NMIBC in patients is to completely remove the tumor and control the unpredictable risk of recurrence and progression to muscle invasion [18] with as little treatment burden and side effects as possible. We know from clinical experience that there are many possible adverse consequences and substantial reductions in HRQoL that differ between treatment options such as chemotherapy versus BCG, induction versus maintenance therapy, and impact of single instillation chemotherapy. However, these have not been well studied or reported, and the key data on PROs are lacking for NMIBC.

The most recent indexed comprehensive review of the literature investigating the impact of NMIBC on HRQoL is over 14 years old and is based on research published between 1966 and 2000 [19]. Our recent systematic review of PROMs in NMIBC (manuscript under review; Rutherford et al) found only 6 out of 19 papers that assessed PROs used a standardized measure. Other limitations of these studies include lack of comparison groups, poor adjustment for baseline physical and psychological functions, failure to distinguish between patients with varying degrees of risk and between treatments in the analysis, and small sample sizes. Consequently, key evidence on the impact of tumor resections, repeated cystoscopies, single chemotherapy instillations, multiple chemotherapy instillations, and BCG therapies on patients’ HRQoL is lacking.

Our review did identify a NMIBC-specific PROM, the European Organisation for Research and Treatment of Cancer (EORTC)—EORTC QLQ-BLS24 questionnaire—developed in 1996. There is no publicly available information detailing its development, and it only recently underwent validation, 18 years after its development (now renamed the EORTC QLQ-NMIBC24) [20]. The validation study had some limitations: it was based on data from a single clinical trial, including only high- and intermediate-risk patients with little information about their treatments; the original content was not reviewed against contemporary treatments (which differ considerably from those in use before 1996 when the original content was developed); modifications to the scale structure were informed by clinical evidence only (ie, no patient perspective); and there is lack of assessment of test-retest reliability and clinical validity (eg, whether the module distinguishes between different risk groups for NMIBC) [20]. The question remains whether the EORTC QLQ-NMIBC24 adequately captures the impact of important symptoms and side effects associated with contemporary treatment for NMIBC.

Therefore, a patient-reported NMIBC-specific symptom index (NMIBC-SI), including possible symptoms and side effects of contemporary treatments for NMIBC, is needed to enable accurate, robust, and clinically relevant assessment of differences in PROs among contemporary treatments and to provide an evidence base for the ongoing improvement of future therapies for NMIBC. Such a measure has been developed based on existing literature and interviews with patients and clinicians [21] and pretested for face validity, comprehensiveness, comprehensibility, and clinical utility through interviews with key stakeholders (patients, urologists, and specialist nurses).

### Aims and Objectives

The overarching aim of this study was to develop and evaluate the NMIBC-SI for reliability, validity, and responsiveness to ensure that it is fit for purpose in clinical research.

#### Psychometric Aims

- To evaluate the feasibility and acceptability of the NMIBC-SI; produce a shorter version, if appropriate, by selecting items that perform best against robust psychometric criteria; examine the legitimacy of summing items into scales; identify legitimate scales and subscales; and test scaling assumptions and scale performance in a large-scale Australian sample (field test 1).
- To psychometrically evaluate the measurement properties of the final version SI, testing for reliability, clinical validity (sensitivity to differences between patient groups and responsiveness to clinically important change), and interpretability of the final version NMIBC-SI in a new large-scale international sample (field test 2).
- To conduct a head-to-head comparison of the new NMIBC-SI with the EORTC bladder cancer module, QLQ-NMIBC24.

#### Clinical Aims (Field Test 2)

- To assess and compare key PROs across the full range of contemporary treatments for NMIBC and over-the-disease trajectory, including acute treatment and 1-year survivorship.
- To compare PROs between patients with low-, intermediate-, and high-risk NMIBC.

## Methods

### Overview of Project Research Design

**Figure 1:**
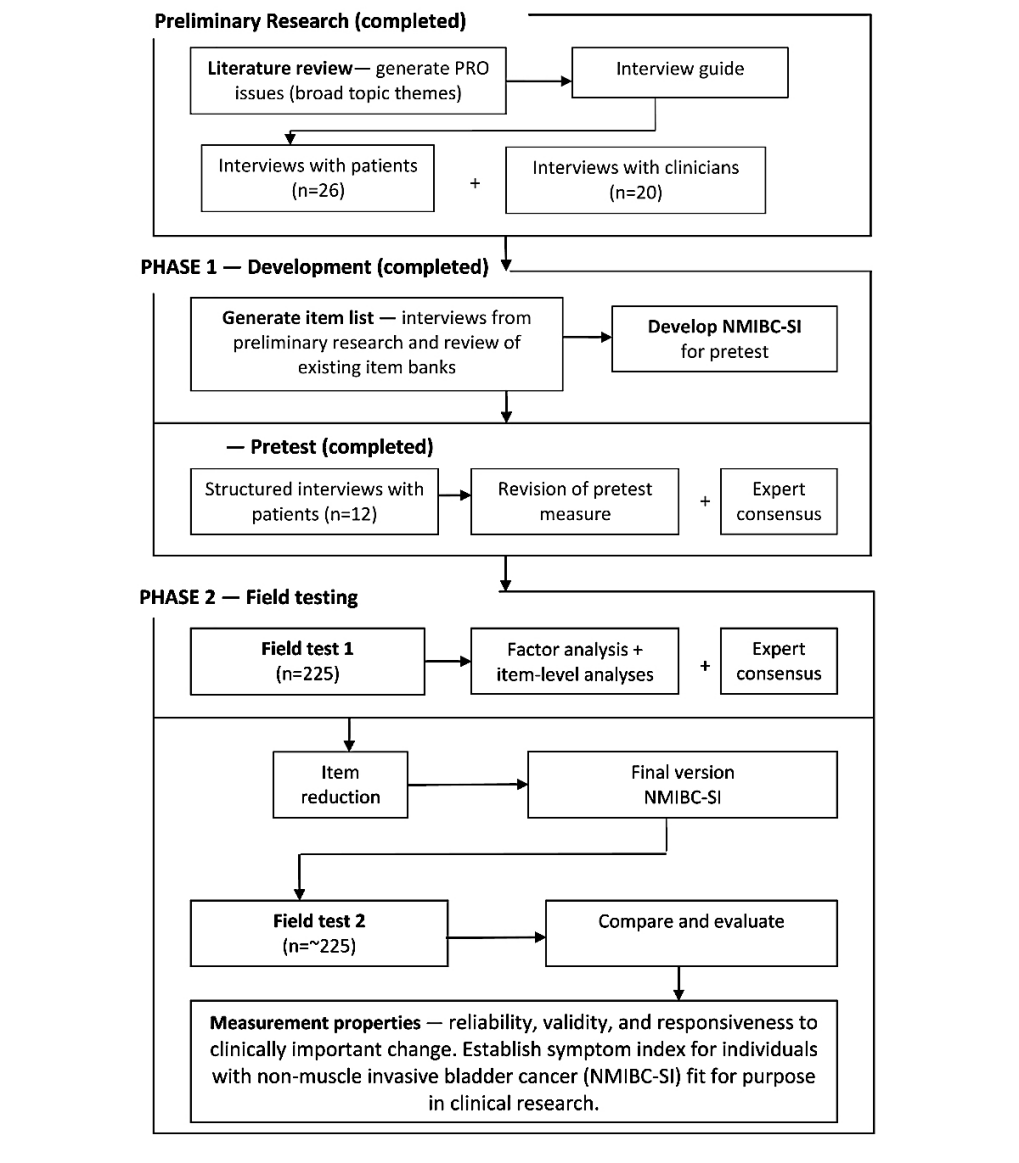
Development and evaluation of the symptom index for individuals with nonmuscle invasive bladder cancer.

This multicenter study was designed to evaluate the psychometric properties of an NMIBC-SI for patients treated for NMIBC. Guidance for developing and validating health outcome measures has been followed to ensure high quality and standardization for the development of the NMIBC-SI [11,22,23]. The guidance recommends that collaboration and expert discussion are sought and utilized through all stages of development, and it proposes distinct phases for the development and evaluation of PROMs. The research design includes 2 phases: (1) development of NMIBC-SI in 2 parts, conceptual framework to generate items and pretesting; and (2) evaluation of NMIBC-SI in 2 parts, a preliminary field test 1 for item reduction and a final field test 2 for psychometric properties (Figure 1). Phase 1 has been conducted, and results are summarized below.

In preliminary research and phase 1 development, a conceptual framework was developed by tapping into 3 sources: (1) a systemic review and narrative analysis of the HRQoL and PRO literature, identifying several local and systematic side effects associated with contemporary treatments for NMIBC (eg, urinary problems, discomfort, and malaise); (2) in-depth qualitative interviews with a sample of NMIBC patients that explored patients’ experience of receiving treatment; and (3) in-depth qualitative interviews with treating clinicians (specialist nurses and urologists) that explored important issues from their perspective. This phase was important to ensure that high content validity was achieved and demonstrated [24]. An exhaustive list of clinically relevant issues (items) was generated from the conceptual framework and patient verbatims [21].

In phase 1 pretesting, further qualitative interviews explored the generated list of issues with patients and clinicians for clarity and overlap and the appropriateness of the NMIBC-SI’s time frame, question stem, and response options. On the basis of information obtained from the interviews, the provisional NMIBC-SI was revised to produce a preliminary version ready for field testing.

The evaluation of the NMIBC-SI is phase 2 of this project. It will be undertaken in 2 parts: preliminary field test 1 (item reduction) and final field test 2 (psychometric properties). The preliminary field test will identify any items with poor psychometric performance for possible elimination. The final field test will be undertaken to evaluate the item-reduced version of the NMIBC-SI for reliability, validity, and responsiveness. Gold standard psychometric methods will be used [11,22,23].

### Phase 2: Field Test 1

The psychometric properties of the NMIBC-SI will be evaluated in 2 field tests, including a preliminary field test (item reduction) to identify items with poor psychometric properties for possible elimination and to identify subscales, and a final field test (psychometric evaluation) to evaluate the reliability and validity of the item-reduced version of the NMIBC-SI. The overall strategy and methods for the psychometric evaluation are based on the methods used to develop and validate PROMs in several other areas of medicine and surgery [24-26].

#### Design for Preliminary Field Test 1 (Item Reduction)

The purpose of the preliminary field test 1 is to produce a short (item-reduced) version of the NMIBC-SI and to undertake an initial psychometric evaluation of the item-reduced questionnaire. This will be done using a cross-sectional study design.

An item reduction strategy developed for evaluation of PROMs in other medical areas [25-27] will be used to: (1) identify items on the provisional version of the NMIBC-SI with poor psychometric properties for possible elimination; (2) conduct a preliminary evaluation of NMIBC-SI subscales; and (3) undertake a preliminary evaluation of the acceptability, reliability, and validity of the item-reduced NMIBC-SI.

Results of the item reduction analyses will be used to develop a shorter version of NMIBC-SI for final psychometric field testing.

#### Eligibility

##### Inclusion Criteria

Adult patients (aged ≥18 years) from participating centers diagnosed with NMIBC, who are able to read and understand English and give their written informed consent will be included in the study. Patients will be recruited while undergoing active treatment (ie, 1 week after tumor resection or intravesical therapy) or when they have completed final treatment for NMIBC within the past week.

##### Exclusion Criteria

Patients will be excluded from the study if any of the following criteria apply:

- They are unconscious or confused.
- They have cognitive impairment.
- They are unable to speak, read, and/or write in English.
- They are diagnosed with muscle invasive disease.
- They are unable to provide informed consent.

#### Sample Size

Approximately 225 NMIBC patients will be required, purposively sampled to ensure representation of patients across the 3 NMIBC risk categories (Textbox 1) [6]. This sample size is based on recommendations for psychometric analyses of new summated scales; 5 to 10 subjects per item are needed to reduce the chance effect of sampling [28,29]. Following this recommendation, a 30-item NMIBC-SI would require a sample between 150 and 300 patients.

#### Recruitment and Consent Procedures

Patients from participating centers who meet the eligibility criteria will be informed about and invited to take part in the study either in person or via an invitation letter. Consecutive patients will be identified and approached to participate by either the named site investigator or the named site nurse. Accrual will be reviewed to ensure that there is balanced representation of patients in all NMIBC categories. A record of those identified as eligible, approached to participate, refusals, consenting patients, and questionnaire returns will be kept for progress monitoring and final reporting purposes (see Data Collection/Assessment section below).

A verbal explanation of the study and a patient information sheet will be provided for patients to consider. These will include detailed information about the rationale, design, and personal implications of the study. Following information provision, patients will have as much time as they need to consider participation. The right of the patient to refuse consent without giving reasons will be respected.

Assenting patients will then be invited to provide informed, written consent on the consent form at the end of the patient information sheet to collect baseline assessment data and to complete the questionnaire. The patient will remain free to withdraw from the study at any time without giving reasons and without prejudicing any further treatment. The original consent form will be filed within the investigator site file or at a designated secure location.

##### Registration

Following informed consent and confirmation of eligibility, participants will be provided with a study ID number and registered to the study.

##### Screening

The participating site staff will complete a log of all patients screened for eligibility. Screening logs will be returned to the University of Sydney.

Using the study ID number as an identifier, information will be collected for all eligible patients, including the age, gender, risk category, and treatment type.

For those who decline to participate in the study, reasons will be recorded.

**Nonmuscle invasive bladder cancer (NMIBC) patient risk groups.**

High-risk tumors

Any of the following:

- T1 tumor
- G3 (high grade, which is a mixture of some G2 and G3) tumor
- Carcinoma in situ (CIS)
- Multiple and recurrent and large (>3 cm) Ta G1G2 tumors (all conditions must be presented at this point); low grade is a mixture of G1 and G2

Intermediate-risk tumors

- All tumors not defined in the adjacent categories (between the category of low- and high risk)

Low-risk tumors

- Primary, solitary, Ta, G1 (PUNLMP, low grade is a mixture of G1 and G2), <3 cm, no CIS

#### Data Collection/Assessment

Study data will be recorded by participating site staff on case report forms and by participants on questionnaire booklets either on paper or electronically. The study data are collected and managed using REDCap electronic data capture tools hosted at the University of Sydney [30].

Assessments will be undertaken as follows:

- Registration and baseline data
- Patient questionnaire booklet

Registration and clinical data will be completed by the clinician or specialist nurse at participating centers. Hard-copy questionnaires will be collected by the clinician or specialist nurse or posted by the patient back to the site. Study ID number coded data will be sent to the University of Sydney for central data management. Individually identifiable data and master lists linking study ID numbers to individual identity will be retained by the participating sites.

#### Registration and Clinical Data

Patients who meet the inclusion criteria and provide informed consent will be registered to this study. Registration and clinical information will be recorded by participating site staff including the following:

- Patient study ID number
- Age
- Gender
- Country of birth
- Marital status
- Living arrangements
- Education
- Risk category (clinical definitive category from medical records)
- Tumor grade
- Tumor stage
- Treatment type (information about which treatment interventions the patient is currently receiving)
- Comorbidity (Charlson Comorbidity Index [31] and history of prostate cancer)
- Name of site staff member completing clinical data
- Confirmation of eligibility

#### Questionnaire Booklet

Participants will self-complete the questionnaire booklet containing the NMIBC-SI, which will be provided to them by the clinician or specialist nurse at participating centers in hard copy or by link to the Web-based version. It is anticipated that completion of the questionnaire may take up to 20 min. Completed hardcopy questionnaires will be entered into REDCap by participating site staff directly or returned to the University of Sydney for data entry.

#### Mode of Questionnaire Administration

Participants will be given a hard copy of the questionnaire or provided with a link to the Web-based questionnaire, depending on their preference.

#### Analyses and Statistical Considerations

To determine whether the NMIBC-SI fulfills fundamental prerequisites for rigorous measurement as defined by established psychometric criteria [32] and the US FDA guidance [11], standard psychometric tests for acceptability, data quality, internal reliability, and factor analysis construct validity will be performed. Table 1, adapted from Gorecki, 2013 [33], presents full details of the tests and criteria used in the psychometric evaluation.

The purpose of the item reduction analysis is to produce a psychometrically robust version of the NMIBC-SI. Standard psychometric tests and criteria, as described in Table 1, will be performed to identify and retain items with strong psychometric properties and eliminate items with poor psychometric properties to produce a shorter, item-reduced version of the NMIBC-SI. These analyses will also evaluate the hypothesized subscales of the NMIBC-SI.

Missing data will not be imputed. The frequency of missing data will be determined, and items with a response rate of <80.0% (180/225) will be investigated.

### Phase 2: Field Test 2—Evaluation of Reliability and Clinical Validity

#### Design for Field Test 2

The purpose of the final field test 2 is to assess the reliability and clinical validity (sensitivity to differences between patient groups and responsiveness to clinically important change) and interpretability of the final version NMIBC-SI in a new large-scale international sample. This phase will be conducted using a prospective longitudinal study design. The outcome will be a patient-reported NMIBC-SI that is reliable and clinically valid, with NMIBC-specific content that complements the more generic HRQoL content of the EORTC QLQ-C30.

#### Eligibility

Adult patients (aged ≥18 years) from participating centers diagnosed with NMIBC, after imaging or flexible cystoscopy but before endoscopic resection, who are able to read and understand English and give their written informed consent will be included in the study.

#### Sample Size

Approximately 225 patients newly diagnosed with NMIBC will be required to provide sufficient subjects for statistical assessment of test-retest reliability [34,35] and clinical validity in terms of both sensitivity to differences between patient groups [36] and responsiveness to clinically important change [37]. See section on Sample Size for Field Test 1 for sample size justification.

**Table 1:** Psychometric tests and criteria.

Property				Definition/test	Criteria for acceptability	
1. Item analysis				Identify items for possible elimination due to weak psychometric performance; assessed on the basis of (1) exploratory factor analysis with principal axis factoring and (2) item- and scale-level analyses	Exploratory factor analysis: items with a factor-loading coefficient ≥0.4 will be retained in each subscale. Applied to all items: (1) missing data <5%; (2) maximum endorsement frequencies <80% (ie, the proportion of respondents who endorse each response category), including floor or ceiling effects <80% (ie, response categories with high endorsement rates at the bottom and top ends of the scale, respectively); (3) evidence of item responsiveness as assessed by significant improvement between baseline and test of cure assessments (field test 2 only)	
2. Acceptability				The quality of data; assessed by completeness of data and score distributions	Missing data for summary scores <20%; normal distribution of endorsement frequencies across response categories (ie, absence of skew, endorsement rates between 0.20 and 0.80); and floor or ceiling effects for summary scores <10%	
**3. Reliability**						
	3.1 Internal consistency			The extent to which items comprising a scale measure the same construct (eg, homogeneity of the scale); assessed by Cronbach alpha and item-total correlations	Cronbach alpha for summary scores ≥.70 and item-total correlations ≥.30	
	3.2 Test-retest reliability (field test 2 only)			The stability of a measuring instrument; assessed by administering the instrument to respondents on two different occasions and examining the correlation between test and retest scores	Test-retest reliability and intraclass correlations for summary scores ≥.70	
**4. Validity**						
	4.1 Content validity			The extent to which the content of a scale is representative of the conceptual domain it is intended to cover; assessed qualitatively during the questionnaire development stage through pretesting with patients, expert opinion, and literature review	Qualitative evidence from patients, expert opinion, and literature review that items in the scale are representative of the construct being measured	
	**4.2 Construct validity**					
		Within-scale analyses		Evidence that a single entity (construct) is being measured and that items can be combined to form a summary score	Confirmatory factor analysis: (1) items with a factor-loading coefficient ≥0.4 and (2) moderate to high correlations between scale scores	
		**Analyses against external criteria**				
			Convergent validity	Evidence that the scale is correlated with other measures of the same or similar constructs; assessed on the basis of correlations between the measure and other similar measures	Correlations are expected to vary according to the degree of similarity between the constructs that are being measured by each instrument. Specific hypotheses are formulated and predictions tested on the basis of correlations	
			Discriminant validity	Evidence that the scale is not correlated with measures of different constructs; assessed on the basis of correlations with measures of different constructs	Low correlations between the instrument and measures of different constructs	
			Known groups differences	The ability of a scale to differentiate known groups; assessed by comparing scores for subgroups that are expected to differ on the construct being measured	Significant differences between known groups or difference of expected magnitude	
5. Responsiveness				The ability of a scale to detect clinically significant change following treatment of known efficacy; assessed by examining within-person change scores before and after treatment and calculating an effect size statistic (mean change score divided by standard deviation of pretreatment scores)	Moderate to large effect sizes (small 0.2, moderate 0.5, and large 0.8 or higher)	

**Table 2:** Patient-reported outcome assessment schedule.

Risk group	Assessed within 3 months before tumor resection	Assessed within 4 to 10 days after the tumor resection	Assessed within 1 month after the end of induction intravesical therapy^a^	Assessed within 1 month before the 1-year cystoscopy (or at early cessation due to adverse events^b^)
High	T1	T2	T3	T4
Intermediate	T1	T2	T3	T4
Low	T1	T2	T3^c^	T4
~n	225	225	225	225

^a^A minimum of 25 participants from each risk group will be asked to complete an additional questionnaire pack 3 to 7 days after T3.

^b^Preferably before cystoscopy.

^c^For the low-risk group, T3 will be 8 weeks after resection.

#### Recruitment and Consent Procedures

Patients will be recruited at diagnosis, after imaging or flexible cystoscopy but before endoscopic resection. Eligible patients will be identified by the clinician investigators and their teams at participating centers, ensuring adequate representation of patients in the 3 risk groups (see Textbox 1) [6]. Patients who meet eligibility criteria will be approached in person and informed about and invited to take part in the study. Consent procedures are similar to field test 1, that is, using patient information sheet and consent form for field test 2.

#### Data Collection/Assessment

##### Registration and Baseline Data

Registration data will be collected as done for field test 1. Baseline questionnaires will be completed before endoscopic resection (Table 2).

##### Follow-Up Data Collection

Participants will complete follow-up questionnaire packs at scheduled follow-up time points (see Table 2). The clinician or specialist nurse at participating centers will be responsible for sending follow-up questionnaires, emails, and reminders to participants. Participants will either be posted a hard-copy questionnaire pack or emailed a link to their follow-up questionnaires, depending on their preference. Participants who complete the Web-based questionnaire pack will be given an option to provide their email address to receive automatic reminders from the REDCap system at follow-up time points. Participants will be advised that email addresses are stored within the REDCap system and used solely for the purpose of sending reminders. REDCap is a secure Web app that runs on the University of Sydney’s servers, ensuring that data stay within the Sydney University data center. The provision of an email address for reminders is entirely voluntary, and participants will be free to change their mind at any point, after which any email address provided will be removed from the REDCap system.

##### PRO Assessment Time Points

Prospective assessment of newly diagnosed patients before and after treatment is required for the responsiveness analysis. It is expected that the NMIBC-SI will detect changes in symptoms due to specific treatments. As risk profile determines treatment schedule, the corresponding schedules of prospective assessments are indicated in Table 2. These time points will also provide PRO data to compare between treatment and risk groups and over time, addressing our clinical aims (see Aims and Objectives section).

In addition to the planned assessments (Table 2), a minimum of 25 participants sampled from each of the 3 risk groups will complete an additional NMIBC-SI 3 to 7 days after the T3 administration to evaluate test-retest reliability. The length of the test-retest interval must be short enough to ensure that clinical change in the NMIBC status is unlikely to occur but sufficiently long to ensure that respondents do not recall their responses from the first assessment. A short test-retest interval is necessary to ensure that stability per se is being evaluated, rather than clinical change during the test-retest interval, which will underestimate the NMIBC-SI’s reliability.

Participants will be sent a hard copy or link to the questionnaires 2 weeks before their scheduled follow-up time point. Up to 2 email or telephone reminders to complete follow-up questionnaires will be made if questionnaires are not completed by the follow-up date. The clinician or specialist nurse at participating centers will be responsible for sending follow-up questionnaires, emails, and reminders to participants unless participants who complete the Web-based questionnaires have provided their email address to receive automatic reminders directly from REDCap. The assessment time windows are indicated in Table 2.

#### Mode of Questionnaire Administration

Participants will be given a hard copy of the questionnaire or provided with a link to the Web-based questionnaire depending on their preference.

#### Questionnaire Booklet

A questionnaire pack containing the NMIBC-SI, EORTC QLQ-C30, and the EORTC QLQ-NMIBC24 will be self-completed by all participants via hard copy or Web.

Participants will complete the item-reduced version of the NMIBC-SI, the QLQ-C30, and the QLQ-NMIBC24 measures to assess construct validity (convergent, discriminant, and known groups; see Table 2). The guiding principle in selecting the validating measures is to include measures that will allow a comparison of NMIBC-SI subscales with measures of similar constructs (convergent validity) and with measures of different constructs (discriminant validity), and to compare NMIBC-SI scores in clinically defined known groups whose HRQoL would be expected to differ.

The QLQ-C30 [38] is a core questionnaire for evaluating the HRQoL of patients participating in cancer clinical trials. It is a 30-item questionnaire with 9 multi-item subscales and 6 single items. It incorporates 5 functional scales (physical, role, cognitive, emotional, and social functioning), 3 symptom scales (fatigue, pain, and nausea or vomiting), and a global health status or HRQoL scale. The single items assess dyspnea, appetite loss, sleep disturbance, constipation, diarrhea, and perceived financial impact of disease and treatment. Ratings for each item range from 1 (not at all) to 4 (very much). The QLQ-C30 is designed to be used across cancer populations and takes about 11 min to complete [38].

The QLQ-NMIBC24 [20] is a 24-item questionnaire for evaluating the HRQoL of patients with superficial (nonmuscle invasive) bladder cancer. The questionnaire is designed to supplement the QLQ-C30 and incorporates 6 multi-item scales and 5 single items. Ratings for each item range from 1 (not at all) to 4 (very much). The scales cover urinary symptoms, malaise, worries about the future, bloating and flatulence, sexual function, and male sexual problems. The single items assess intravesical treatment issues, sexual intimacy, sexual enjoyment, risk of contaminating partner, and female sexual problems.

All measures will be administered in the same order. It is anticipated that completion of questionnaire packs may take up to 20 min.

#### Analyses and Statistical Considerations

Analyses will evaluate the measurement properties of the final version of the NMIBC-SI, using psychometric tests described for field test 1 (Table 1). Additional tests for reliability (test-retest: correlations for summary scores ≥.70), clinical validity (in terms of sensitivity to groups known to differ clinically and responsiveness to clinically meaningful change over time [37]), and estimation of minimal important difference (interpretability) [39] will be undertaken. Evaluation of subscales will be determined by factor analysis and against external criteria (between-group validity: convergent, discriminant, and known group differences validity). To evaluate convergent and discriminant validity, we will compare NMIBC-SI scales with the scales within the EORTC QLQ-C30 and QLQ-NMIBC24 measures. NMIBC-SI scores for patients by risk and treatment groups will be compared to evaluate known group differences. Risk groups are defined as low, intermediate, and high (see Textbox 1) [6]. Treatment groups are defined as follows: (1) cystoscopy alone or TURBT, (2) course of intravesical chemotherapy, and (3) course of intravesical BCG. We hypothesize that low-risk patients will have lower levels of treatment-related problems at follow-up time points compared with high-risk patients, who have more intensive treatments. Factor analysis, together with the results of other item-level analyses described in Table 1, will be used to investigate hypothesized subscales.

Analyses of the clinical aims will include: (1) evaluation of PRO changes over time, from diagnosis to peak treatment and at 1 year; (2) comparison of PROs between the 3 risk groups at each time point; and (3) head-to-head comparison of the relative discriminatory ability of the NMIBC-SI and the EORTC QLQ-NMIBC24. The pretumor resection assessment (T1) will be at diagnosis and is considered our baseline assessment. Patients are not expected to have any treatment-related problems at this point. The subsequent 3 time points (Table 2) are intended to capture short-term, intermediate, and long-term levels of problems, and each will be compared with baseline (T1).

## Results

### Development of NMIBC-SI

A patient-reported NMIBC-SI, including all symptoms and side effects associated with contemporary treatments for NMIBC, has been developed based on a conceptual framework of PROs important to patients with NMIBC and their managing clinicians [21].

The conceptual framework was developed by utilizing 3 sources (see Figure 1): (1) a systematic review and narrative analysis of the PRO literature relevant to NMIBC identified several local and systemic side effects associated with contemporary treatments for NMIBC (eg, urinary problems, discomfort, and flu-like symptoms); (2) in-depth qualitative interviews with a sample of NMIBC patients explored patients’ experience of receiving treatment; and (3) in-depth qualitative interviews with treating clinicians (specialist nurses and urologists) explored important issues from their perspective. These aspects were important to ensure that the new NMIBC-SI had high content validity [24]. An exhaustive list of clinically relevant issues (items) was generated from the conceptual framework and patient and clinician verbatims.

### Pretesting of the NMIBC-SI

The draft version of the NMIBC-SI was pretested using structured interviews with key stakeholders (patients and clinicians), testing for face validity, relevance and comprehensiveness of content, comprehensibility, and clinical utility. Specifically, clarity and overlap of items and the appropriateness of the NMIBC-SI’s time frame, question stem, and response options were explored. On the basis of information obtained from the interviews, the provisional NMIBC-SI was revised to produce a preliminary version ready for field testing.

The NMIBC-SI was designed to complement and be administered alongside the EORTC QLQ-C30 generic cancer questionnaire [38] (which includes core domains of functioning and HRQoL, fatigue and general pain but no NMIBC-specific symptoms).

### Item Reduction of the NMIBC-SI: Field Test 1

The field testing phase is planned over 3 years. Recruitment for field test 1 commenced in February 2017 in 9 Australian centers. Recruitment for field test 2 is planned to commence in January 2018 in the same 9 Australian centers that participated in field test 1 plus an additional 10 international centers (2 in New Zealand, 4 in the United States, 2 in Canada, and 2 in Europe) following the universal approach to using the same language in different countries [40]. These 19 centers include both public and private hospitals and treatment clinics. Final results are expected to be published in 2019. The NMIBC-SI will be freely available for use via registration.

## Discussion

This study protocol contains detailed methods to be used across 19 international sites, including both public and private hospitals and treatment clinics that treat patients diagnosed with NMIBC. Field test 1 is a cross-sectional study and includes a sample of patients with NMIBC on active treatment. Field test 2 is a longitudinal study and includes a sample of newly diagnosed patients to enable assessment of possible treatment effects as well as responsiveness of the NMIBC-SI. No patients recruited for field test 1 will be included in field test 2. Phase 2 in the development of the NMIBC-SI will enable a comprehensive evaluation of its reliability, validity, and responsiveness to ensure that it is fit for purpose in clinical research and provides an evidence base for the ongoing improvement of future therapies for NMIBC.

Following evaluation, our NMIBC-SI will be suitable for use with English-speaking patients, diagnosed and treated for NMIBC in Australia, New Zealand, the United States, Canada, and the United Kingdom. Cross-cultural and language translations are planned following development and evaluation of the English version.

## Appendix

Multimedia Appendix 1 Peer Review Report.

## Abbreviations

BCG: Bacillus Calmette–Guérin
EORTC: European Organisation for Research and Treatment of Cancer
HRQoL: health-related quality of life
NMIBC: nonmuscle invasive bladder cancer
NMIBC-SI: symptom index for individuals with NMIBC or NMIBC-specific symptom index
PRO: patient-reported outcome
PROM: patient-reported outcome measures
SI: symptom index
