# Supplementary material for: Psychometric Evaluation of a Patient-Reported Symptom Index for Nonmuscle Invasive Bladder Cancer: Field Testing Protocol
Source: JMIR Res Protoc. 2017 Nov 8;6(11):e216. doi: 10.2196/resprot.8761 (PMC5700405; doi:10.2196/resprot.8761)
Supplement: Multimedia Appendix 1 [file resprot_v6i11e216_app1.pdf]

Research Office
Kolling Building, Level 13
Royal North Shore Hospital
St Leonards NSW 2065
Tel (02) 9926 4590 Fax (02) 9926 6179

NSW GOVERNMENT | **Health** Northern Sydney Local Health District

15 August 2017

A/Prof Manish Patel
Western Clinical School
Westmead Hospital (C24)
University of Sydney NSW 2006

Dear Manish

NSLHD reference: RESP/16/219
Title: Psychometric evaluation of a Patient-Reported Symptom Index for Non-Muscle Invasive Bladder Cancer: Phase 2 field testing
HREC reference: HREC/16/HAWKE/329

Thank you for submitting a request for an amendment to the above project, dated **29 June 2017, received 10 July 2017.** This was considered by the Northern Sydney Local Health District (NSLHD) Human Research Ethics Committee (HREC) at its Executive meeting held on **2 August 2017.** This HREC has been accredited by NSW Ministry of Health as a Lead HREC under the model for single ethical and scientific review and Certified by the NHMRC under the National model for Harmonisation of Multicentre Ethical Review (HoMER). This lead HREC is constituted and operates in accordance with the National Health and Medical Research Council's *National Statement on Ethical Conduct in Human Research* and the *CPMP/ICH Note for Guidance on Good Clinical Practice*. No HREC members with a conflict of interest were present for review of this project.

I am pleased to advise that the documents reviewed and approved at the meeting were:

Document	Version	Date
Study Protocol	3	1 June 2017
Master Participant Information Sheet and Consent Form – Field Test 2	3	1 June 2017

For multi-site projects reviewed by the HREC after 1 July 2007 a copy of this letter must be forwarded to all Principal Investigators at every site approved by NSLHD HREC for submission to the relevant Research Governance Officer along with a copy of the approved documents.

Should you have any queries about your project please contact the Research Office, Tel: 9926 4590, email NSLHD-Research@health.nsw.gov.au . The HREC Terms of Reference, Standard Operating Procedures, *National Statement on Ethical Conduct in Human Research* (2007) and the *CPMP/ICH Note for Guidance on Good Clinical Practice* and standard forms are available on the Research Office website: http://www.nslhd.health.nsw.gov.au/AboutUs/Research/Office

Please quote NSLHD reference **RESP/16/219** in all correspondence.

Yours sincerely

Monique Macara
Research Ethics Manager
Northern Sydney Local Health District

cc. Margaret-Ann Tait

RESD/17/5931
